# Life’s Order, Complexity, Organization, and Its Thermodynamic–Holistic Imperatives

**DOI:** 10.3390/life2040323

**Published:** 2012-11-13

**Authors:** Richard Egel

**Affiliations:** Department of Biology, University of Copenhagen Biocenter, Ole Maaløes Vej 5, DK-2200 Copenhagen, Denmark; E-Mail: regel@bio.ku.dk; Tel.: +45-4589-3746; Fax: +45-3532-2128

**Keywords:** origin of life, far-from-equilibrium thermodynamics, agglutinative hydrophobic interactions, phase-separated catalytic microspheres, metabolic-replicative hypercycles, hyper-coupling between separate sequence spaces

## Abstract

*In memoriam* Jeffrey S. Wicken (1942–2002)—the evolutionarily minded biochemist, who in the 1970/80s strived for a synthesis of biological and physical theories to fathom the tentative origins of life. Several integrative concepts are worth remembering from Wicken’s legacy. (i) Connecting life’s origins and complex organization to a preexisting physical world demands a thermodynamically sound transition. (ii) Energetic ‘charging’ of the prebiosphere must precede the emergence of biological organization. (iii) Environmental energy gradients are exploited progressively, approaching maximum interactive structure and minimum dissipation. (iv) Dynamic self-assembly of prebiotic organic matter is driven by hydrophobic tension between water and amphiphilic building blocks, such as aggregating peptides from non-polar amino acids and base stacking in nucleic acids. (v) The dynamics of autocatalytic self-organization are facilitated by a multiplicity of weak interactions, such as hydrogen bonding, within and between macromolecular assemblies. (vi) The coevolution of (initially uncoded) proteins and nucleic acids in energy-coupled and metabolically active so-called ‘microspheres’ is more realistic as a kinetic transition model of primal biogenesis than ‘hypercycle replication’ theories for nucleic acid replicators on their own. All these considerations blend well with the current understanding that sunlight UV-induced photo-electronic excitation of colloidal metal sulfide particles appears most suitable as a prebiotic driver of organic synthesis reactions, in tight cooperation with organic, phase-separated, catalytic ‘microspheres’. On the ‘continuist *vs*. miraculist’ schism described by Iris Fry for origins-of-life considerations (Table 1), Wicken was a fervent early protagonist of holistic ‘continuist’ views and agenda.

## 1. Memorial Introduction

### 1.1. Understanding Life as Earth’s Response to Thermodynamic Principles: Surfing at Crested Waves on Cosmic Gradients

This essay was developed from my belated acquaintance with Jeffrey Wicken’s seminal works on early evolutionary energetics. So I begin this introduction in his memory. It is mainly from Wicken’s writing that I have learned about some key considerations by Simon Black. Moreover, through Wicken’s views I began to look at Sidney Fox’s prolific work on “*proteinoid microspheres*” (Section 3.1.) in a new light.

### 1.2. Jeffrey S. Wicken (1942–2002)

A quarter century ago, an American biochemist turned *theoretical biologist*, Jeffrey Stephen Wicken, compounded his insights and views about “*Evolution, Thermodynamics, and Information*” in a seminal book, with stakes as high as “*extending the Darwinian program*” [1]. Yet, in 2002, the scientific community took but little notice of Wicken’s passing away in virtual seclusion. Closest to a laudatory obituary, Eric Schneider and Dorian Sagan, authors of another book [2], repeatedly acknowledged Wicken’s name among “*preceding giants*” in the field of *nonequilibrium thermodynamics*. “*The late Jeffrey Wicken laid the theoretical groundwork for ourapplication of thermodynamics to much of biology*” ([2], p. xix). Moreover, the only personal reminiscence published of Wicken’s life and whereabouts is embedded in their book as follows.

“*To* [the] *developing pantheon of nonequilibrium thinkers, we must now add Wicken* [1]*, who dares to claim that it is only because of the second law of thermodynamics that life exists at all. The late Jeffrey Wicken completed some of Lotka’s and Schrödinger’s unfinished thoughts on the thermodynamic nature of life. Wicken persuasively argued that the second law is not just compatible with life but instrumental in its origins and evolution. Wicken, the lecturer, was a caged lion. With a black eye patch (the result of a childhood accident) he would pace, spin on his heels to the board to write an equation that baffled his audience. His intensity was notable as his discourse jumped from the early Greek philosophy of Zeno and the Eleatics or deficiencies in the Newtonian paradigm to the bonding strength of peptides. He was selected by students year after year as a finalist for best lecturer at Behrend College of Pennsylvania State University in Erie. A biochemist by training, Wicken had turned to theoretical biology and thermodynamics because his small college lacked research facilities in molecular chemistry. Beginning in 1978, he published thirty-five papers and a book in twelve years. Although hampered by alcoholism in his later years, he was, in our estimation, a major contributor to ideas of nonequilibrium thermodynamics and biology. Wicken unveils the connections between autocatalysis—linked, self-perpetuating networks — and thermodynamics, showing them to be woven from a single cloth, the same tapestry whose embroidery includes the origins of life*”.

([2], p. 105)

**Figure 1:**
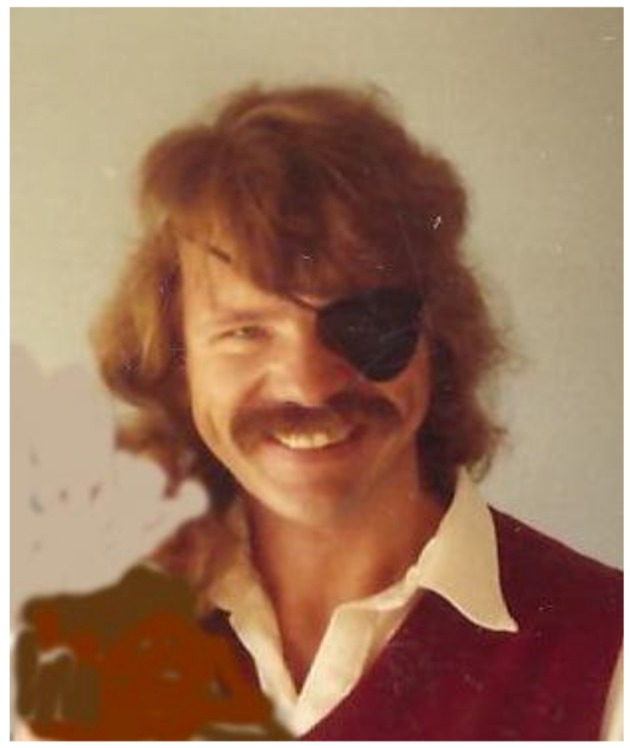
Jeffrey Wicken (ca. 1990)—a smiling twinkle in his eye, which luckily was spared from an unfortunate childhood accident. (Photograph: courtesy Jessica Wicken, who has slightly retouched the background to the left, concealing another person unrelated to this scientific paper).

In his family, from the height of his prime (Figure 1), Jeff Wicken is still kindly remembered as a very handsome, funny and playful person, a terrific bridge player, too, as well as being a serious scientist—having an extraordinary mind, quick to grasp changes and new concepts.

In addition to Erwin Schrödinger and Alfred Lotka of the preceding quote, as well as other influential predecessors mentioned elsewhere in that book, David Layzer’s views on growth of order in the universe, and the cosmological ramification of biological evolution in particular, should serve as further benchmarks for comparison with Wicken’s elaborations and achievements [3,4]. As for myself, I happened to come in from a sideline rather recently, trying to utilize my experience as molecular geneticist more broadly, as to its bearing on scientifically motivated conceptions about tentative origins of life on Earth [5]. Upon reading Wicken’s book [1] in hindsight now, I am indeed attracted by various lines of argument in his discourse. Three characteristics, in particular, appeal to my personal views and inferences about the distant prebiogenic transition period.

- Wicken’s keen acuity in the choice of appropriate wording and conceptions;
- His integrative bracing and embracing of disparate yet highly complementary concepts;
- His determined aiming at ultimate connections between the biosphere’s tentative emergence and fundamental principles of the physical world;
- And not the least, his decidedly holistic views on the nature of life and its emergence—a remarkable trait not equally expressed in preceding origin-of-life research.

In this essay, therefore, I primarily discuss Wicken’s drive and insights from origins-of-life perspectives. The integrative objective behind his writing clearly was “*to show the coherence of life with the rest of nature*” [6]. The main components for such connectivity aim at rationalizing life in terms of sound thermodynamic ramifications, regarding both the organization of living matter as such (Section 2.2.) and the emergence of Darwinian evolution (Section 3). The main importance of these contributions in his time was pointed out by Daniel R. Brooks and coworkers from the ecological perspective, “*Wicken’s concern has been primarily with the origins of life, and the role of thermodynamic interchanges with the environment in the evolution of life*” [7]. Bruce Weber, too, recognized his systems-minded integrative approach, “*Jeffrey Wicken specifically sought to extend the synthesis through use of non-equilibrium thermodynamics, particularly as applied to the problem of the origin of life*” [8]. As for an ongoing debate about the most likely preconditions for the beginnings of prebiotic evolution and natural selection—of ‘*metabolists*’ *vs*. ‘*geneticists*’ [3]—Jeffrey Wicken’s name is called upon as a dedicated protagonists of a *metabolism-first* scenario, assuming the primacy of an energized prebiosphere with system-wide protometabolic interactions, from which genetic replicators had to emerge later on [1]. From this pivotal bifurcation step onward, he saw proto-life as evolving in holistic duality, comprising functional catalyst systems in parallel continuity with informational memory depositories of the genetic kind.

### 1.3. Simon Black (1917–2008)

Unlike Jeffrey Wicken, the theorist, Simon Black was an experimental biochemist throughout his active professional career, which he spent on various enzyme activities at NIH, Bethesda, MD, from 1952 to his retirement in 1993 [9]—with merely two theoretical excursions into the tentative energetics of origins of life on Earth [10,11]. In particular, his motivated theses that hydrophobically driven synthesis of prebiotic peptides lay at the heart of primal self-organization [10] and “*that life arose from inanimate matter for thermodynamic reasons, and that the fundamental criterion for survival is the capacity of organisms to accelerate the dissipation of free energies in organic chemical-water systems*” [11] deserve to be mentioned here for their influence on Wicken’s evolutionary theories. Among the ~15 articles listed by Google Scholar as citing Black’s latter paper, in fact, Wicken has authored five of these.

## 2. Wicken’s Response to Schrödinger’s Dual Challenge

### 2.1. Schrödinger’s Cause in a Nutshell

In February 1943, Jeff Wicken was merely a toddler of age, whilst Europe struggled in turmoils of war. That is when Erwin Schrödinger—the illustrious émigré, ‘*Out of Austria*’, a theoretical quantum physicist of the highest reputation, and a natural philosopher, too—gave a prominent lecture series at Trinity College in Dublin, Ireland. Subsequently, as derived from these lectures, a charmingly streamlined yet highly ambitious booklet literally changed the course of modern biology—striving for conceptual answers to the ultimate query, “*What is Life?*” [12]. It is not so much that this booklet told traditional biologists what to do next or which way to turn. Rather, its major impact was to inspire a generation of dedicated physicists and physical chemists to set their wits on the peculiar *orderliness* of living matter, as contrasted with anything hitherto examined in an ordinary physics laboratory. 

Actually another physicist, Max Delbrück, had already prepared the grounds for such a transition, by focusing on the physical induction of mutations [13], as well as by advocating bacterial viruses (bacteriophages) as minimized model packages of genetic material. With due reference to Delbrück’s work and conceptions, Schrödinger put the uniqueness of genetic determinism at the center of his principal considerations, even though virtually nothing was known to him or others about the biochemical mechanisms behind the genetically influenced phenomena. To connect the working of a living organism to physical laws in a modern sense, it is necessary to understand its organizational complexity in terms of atomic statistics, stochastic fluctuations, and regular flow patterns of matter coupled to energy degradation. This is the domain of statistical thermodynamics.

As the central hub of his booklet ([12], chapters 6 and 7), Schrödinger points out “*two ways of producing orderliness*” in living systems, “*the ‘statistical mechanism’ which produces ‘order from disorder’ and the new one, the ‘order-from-order’ principle*”. It is mainly in pursuit of the latter principle that modern molecular biology has since progressed and made its many fundamental breakthroughs. 

Well before the involvement of nucleic acids in genetic affairs was widely accepted, or the double-helical structure of genomic DNA itself had been established, Schrödinger was captivated by the uniqueness and stability of the “*governing atoms*” in a germ cell, which resided in a minuscule segment of but a single chromosome fiber per haploid genome. Such *genes* could retain recognizable identity over centuries or more, but also could change the configurational state in saltatory *mutations*, the “*quantum jumps*” of genetics, which in turn could be triggered by energy transfer in X-ray induced ionization events. Presciently indeed, he could only conceive of such configurational complexity in chromosome fibers as being “*compressed in the miniature code*” of “*aperiodic solids*”. In his generalizing description, Schrödinger juxtaposed two different groups of categories by liberally ‘equalizing’ certain peculiar states of matter:

“*molecule = solid = crystal*” *vs.* “*gas = liquid = amorphous*”.

From this lumping together of partly overlapping concepts, the phrasing as “*aperiodic crystals*” is most often referred to in subsequent quotes, although the implicit interpretation as “*aperiodic molecules*” should sound more familiar in terms of modern molecular biology. So much for the “*order-from-order principle*” of Schrödinger’s two-pronged approach to characterizing the generation of orderliness in living matter. This aspect has since been elucidated by the concepts of DNA replication, transcription into RNA and translation in ribosome-mediated protein synthesis. The other side of the medal, however—creating “*order from disorder*”—is more difficult to comprehend, especially when it comes to early beginnings of the biosphere.

In Schrödinger’s own words, the delicate organization of living organisms is maintained by “*concentrating a ‘stream of order’ on itself*”, by “*extracting ‘order’ from the environment*”, or still more figuratively, by “*feeding on negative entropy*”. Plants, in particular, “*have their most powerful supply of ‘negative entropy’ in the sunlight*” [12]. Through the networks of metabolic reactions, high-quality energy (~ low entropy) from sunlight or organic foodstuffs is partly degraded to low-quality energy of waste products and heat (~ high entropy/disorder). Life’s secret is coupled to the pivotal other part of the high-quality potential energy consumed, which, instead of being degraded to waste or heat immediately as well, is utilized and invested in growth and reproduction, so as to generate ever more living matter of self-similar organization. Life has evolved, therefore, as an effective beam splitter, using a maximal fraction of incoming sunlight (or geochemical energy) for synthesis of semi-stable organic matter and a corresponding minimum fraction for instant conversion into ambient low-temperature heat. In such general terms, therefore, the theoretical physicist is satisfied that existing life as such does not violate the universal laws of thermodynamics. 

On the other hand, just as Darwin refrained from discussing the ultimate origin of the very first living species from non-organismic precursors, Schrödinger did not at all address the ultimate beginnings of his biogenic “*order-from-order*” and “*order-from-disorder*” principles. This is where Wicken and others stepped in to pursue those farther goals of deriving initial life-like organization from primordial chaos. In a historical perspective, though, also Schrödinger’s quest for life’s characteristics can be regarded as culminating from a long tradition [14], of which the oscillating “*genes vs. protoplasm/metabolism*” debate is still reverberating (see below).

### 2.2. Wicken’s Dealing with the Emergence of Orderliness from Thermodynamic Principles

During his most productive decade at Penn State Erie Behrend College, Jeffrey Wicken was at no position to compete with mainstream biological chemists to polish up the experimental details of current molecular biology, which now constitute the “*order-from-order*” frame of Schrödinger’s dual vision about the essence of life. On the other hand, the enigmatic challenge of pursuing the “*order-from-disorder*” aspect to its tentative beginnings suited Wicken’s talents well. His integrative spirit and synthetic abilities have made a significant difference indeed to further understanding. Thus, Wicken was about first to venture a conceptual synthesis of Layzer’s and Lotka’s theorems, which gathered thermodynamic arguments from, respectively, cosmological and ecological perspectives.

David Lazer linked unidirectional entropy-generating processes in closed systems and information-generating processes in certain open systems to the cooling down of an ever-expanding universe [15,16]. In quite general terms, of course, such considerations would also provide for the emergence and sustenance of life, but Layzer himself did not engage in detailed specifics on how to accomplish this—“*still a mystery*”—transition [17]. In terms of overall source–sink relationships, the hot radiating sun is constantly losing energy to the cold voids of outer space. Yet the specifics of life’s emergence must have depended on how local earthly conditions interfered with this superior cosmic gradient.

Alfred Lotka, on the other hand, elaborated on Boltzmann’s elementary conjecture that contention for available energy be the superior objective of evolution in organic systems [18]. Natural selection will operate to preserve and increase such organisms and entire ecosystems that enlarge the total energy flux through the system. In general, Lotka viewed living organisms as “*energy transformers*” [19]—be it of a net accumulating kind, such as plants, or mainly dissipative engines, such as animals. This dual distribution into largely segregated types can also be described as “*autotrophic anabions*” *vs*. “*heterotrophic catabions*” [18]. Moreover, inasmuch as evolving organisms comprise multitudes of similar units—many kinds occurring in large numbers—this invites statistical treatment and the development of a statistical mechanics of a new kind, as opposed to ordinary reversible collisions of simple material particles. “*The units in the new statistical mechanics will be ‘energy transformers’ subject to irreversible collisions of peculiar type—collisions in which ‘trigger action’ is a dominant feature*” [19]. These early papers are considered to mark the beginning of *ecological thermodynamics* [20]. To be more comprehensive, though, living organisms are also *information processors* ([1], p. 31); they come into being and maintain themselves through self-informed and self-organized means.

As Wicken sums up in his Preface, “*This book is about the emergence and evolution of life. The two are rarely spoken of in consecutive breaths, and have never been brought into a single conceptual framework*” ([1]: p. v). Paving the way for such a merger, and calling upon “*philosophy’s critical function*” [21], he points at the “*prevailing autonomy-of-biology posture*” throughout contemporary evolutionist theory [22,23] as a severe impediment to a productive dialogue with the physical sciences, and with thermodynamic considerations, in particular. To be sure, the related notion that physical theorems appeared irrelevant to biological organization was quite evident to Immanuel Kant and Charles Darwin, when merely mechanical principles comprised their contemporaries’ science of classical physics. Yet, modern physics has moved a lot and is moving further still, which has already proved its worth throughout functional biology in many ways. So a constructive dialogue between physics and evolutionary biology, too, should no longer be considered an altogether illusionary goal—not least when it comes to discussing the tentative origins of life, and for that matter, the very beginnings of Darwinian evolution. 

Getting to grips with thermodynamic terms appropriate and adequate for life’s emergence is central to the formidable challenge. Not meeting this demand would indeed alienate thermodynamics as anunwelcome stranger, but “*this ‘uninvited guest’ will not go away, nor will the biological evidence to the contrary notwithstanding*” [24]. Wicken, in fact, welcomes this enigmatic stranger as a key mediator in the initial gathering of biogenic configurational information. In a seminal series of related papers [25,26,27] he proposes to formalize the different aspects of Schrödinger’s ‘*orderliness*’ of life. What is it, specifically, that renders living organisms capable of “*extracting ‘order’ from the environment*” and, in turn, exporting disorder/entropy/heat to the surroundings [12]?—Somehow, at any rate, the emergence, maintenance and propagation of the living state must become comprehensible within the constraints of the famous (irreversible) *second law of thermodynamics*.

To clarify terms and to emphasize different levels of ‘orderliness’, Wicken argues along the following lines. First of all, living beings are not simply ‘ordered’ systems in any classic-mechanical or mechanistic sense, but they are characterized by hierarchical complexity and delicate internal *organization*, as based on multiple process-oriented and structure-mediated—kinetic—interactions. Next, Wicken scrutinizes the second law for what it actually means for biogenic conditions [25]. Notably, its widest known imposition of complete disordering and randomization along the ‘*thermodynamic arrow of time*’ only prevails under very minimalistic presumptions, as assumed for a rarified gas in a hermetically closed and isolated system, where elastic ‘billiard ball’ collisions are the only type of interaction allowed amongst the individual particles. Yet, none of this applies as such to anything on Earth. More realistically, different ‘*arrows of time*’ are crossing each other during cosmological evolution of open and energy-absorbing systems. Most importantly, the overall cooling effect of the expanding universe facilitates progressive accretion of elementary particles, atoms, molecules, crystals, and so forth [15,16]. Notably, the less stringently definable complex structural assemblies occurring at various levels by agglutinative hydrophobic interactions in water-permeated living matter, as recognized by Simon Black [11], likewise belong to this category of aggregational consolidation, albeit of an inherently dynamic and kinetic kind.

To comprehend the impact of this ‘crossing of different arrows’ on the range and scope of the second law, as applied to the statistical theory of chemical reactions, Wicken introduces separate terms for thermal and configurational contributions, thus allowing *configurational information* to increase (as a measure of material order), together with thermal dissipation of part of the energy flow to the surroundings (net decrease in order overall) [25]. This approach ties both constructive and destructive (randomizing) principles—respectively *information* and *entropy*—into a common formalism; for the latest thinking about the enigmatic relatedness of *entropy* and *information* as complementary concepts, see the informative essay of Robert Ulanowicz [28]. Yet, combining configurational and dissipational terms in a composite formalism does not as such explain the directional progression of prebiotic evolution. How does structural complexification feed back on energy flows in the dissipation of preexisting energy gradients? 

To summarize his general views, Wicken considers thermodynamics and evolution comparable in their overall independence of kinetic mechanisms. Such overall superiority to mechanistic particularities notwithstanding, some operational means are, of course, required to confer any changes accommodated by thermodynamic or evolutionary principles.

“*Thermodynamics and evolution both exemplify the two-tiered complementarity of how and why. Like adaptation, the second law is mute on the subject of mechanism. It rather expresses the teleomatic drive to randomize matter and energy in probability space—a drive that exists independently of kinetic mechanisms, but for whose attainment kinetic mechanisms are required. Thermodynamic explanations do not concern themselves with those microscopically-reversible mechanisms themselves, but with the macroscopic conditions—the thermodynamic gradients—which give them direction. ... The analogy between the teleomatic ends of thermodynamics and the adaptational ends of evolution is more than skin deep. Organisms are informed dissipative structures, maintaining organization by processing energy. Self-production and reproduction through informed energy utilization is the general condition of all adaptation. Given this, life can be most consistently regarded as having emerged as informed autocatalytic systems able to exploit thermodynamic gradients*”

[21]. (The implications of the term ‘teleomatic’ are explained at the end of Section 3.2.)

More mechanistically, when Wicken discussed the potential of thermodynamic ‘*driving forces*’ in prebiotic evolution, he briefly refers to Black’s implication of agglutinative hydrophobic forces in organic self-assembly ([1], p. 81). Simon Black, in turn, is more elaborate on this issue: “*the free energy available for a process is often called its driving force. ... Polymers and more highly organized structures cannot form without a channeled input of energy, and the energy processing cannot occur without specifically organized structures. ...* [In a rate-regulating feedback loop] *the products of self-assembly facilitate the first series of steps through which they are formed. A logical inference is that the self-assembled structures came into existence first, and the feedback loop was selectively evolved because it facilitated their further accumulation as an autocatalytic function. ... Could* [the formation of linear polymers from monomers] *have been spontaneous when it was billions-fold slower? ... Evolution should then have begun as a spontaneous self-assembling process … an aggregative discharge of hydrophobic energy. But why did the simple first structures give rise to infinitely more complex organisms? The answer is that organisms discharge* [some] *energy a billion billion times faster*” [11]. The hydrophobic component of structural self-organization is clustered around a trinity of topological archetypes: cores, scaffolds and envelopes, as further elaborated on in Section 4.2..

When it comes to the discharging of large-scale energetic gradients in a structurally heterogeneous environment, such discharge is not necessarily a smooth affair, since part of the downhill energy flow can be diverted into temporary reservoirs at various intermediate levels. It is one of life’s most basic activities to utilize accessible energized reservoirs of their environment for metabolic purposes, as well as to build up intermediary reservoirs of its own, which subsequently function as internal storage for back-up and future use. Photoautotrophic cells, for example, can only dissipate sunlight energy directly during daytime; thus charging their internal batteries allows them to spread out dissipation of free energy sufficiently long to survive the night or even longer periods of darkness. With this in mind, Wicken assumes that some “*energetic charging of the prebiosphere*” was a crucial thermodynamic prerequisite for life’s emergence ([1], p. 71). Furthermore, the generation of coherent chemical complexity was likewise necessary to channelize that energy in self-maintaining and self-proliferative dissipative processes. The energetic charging and some accompanying buildup of proto-metabolic chemical complexity must have preceded the emergence of genetic replicators as fortifying agents from stochastically oligomerized and differentially aggregated organic compounds.

The following passages from early papers are quite suited to round off this overview on Wicken’s general ideas. “*The generation of molecular organization is the subject of molecular evolution; randomizing or disordering processes constitute the subject matter of irreversible thermodynamics. Clearly, if the two are to be commensurable, an intimate connection must be established between randomizing processes and the generation of organization. The randomizing directives of the second law are not only commensurable with the generation of organized complexity in chemical systems, but are, in fact responsible for this development*” [25]. “*The Second Law acts to dissipate order, not organization; indeed the latter tends to accumulate at the expense of the former*” [26].

## 3. Wicken’s Extension upon Darwinian Principles

### 3.1. Wicken’s Dealing with Molecular Neo-Darwinism

“*The ontogenesis of biological organization is powered by the thermodynamic arrow, but informed by genetic sequences*” [27]. The dual imperatives of this quote from Wicken’s writing impose a conceptual hierarchy on Schrödinger’s ordering principles in biological organization, setting the metabolic generation of “*order from disorder*” prior to—and causally superior to—the genetic propagation of “*order from order*”. In a subtle yet definite manner, this significant twist subjects the powerful controls of genetics—pervading about every aspect of modern life—under the overarching umbrella of even more imperative directives of geo/biochemical energy fluxes, as empowered by the dissipation of life-supportive energetic gradients.

In taking and defending such an unconventional stance, Wicken was bold enough to argue against quite influential currents in mainstream opinion of his time. In fact, the undeniable success of experimental molecular biology—in revealing the informational roles of nucleic acids in modern cells—had focused attention mostly on the tentative emergence of replicable agents, which has long been regarded to be the crucial factor in origin-of-life considerations. A prominent series of papers highlighted the prevalence of that assumption—Manfred Eigen’s elaborations on various aspects of his *replicative hypercycle model* as the origin of natural self-organization in general [29,30,31,32,33,34,35], with substantial input from Peter Schuster in the central trilogy [31,32,33], as well as other coworkers. Jeffrey Wicken subjected major conceptions of that model to a rigorous critique [36].

Quite generally, *hypercycle* is to denote any chain of chemical reactions that can be arranged in a circular scheme, provided that at least one of the constituent reaction steps itself comprises a cyclic succession of intermediate reactions [29,31], and energetic input at one or more steps keeps the entire cycle running. A *hypercyclic system* is a *closed loop of catalytic couplings* [37]. To begin with, and in the most general framing of Eigen’s catchy term of ‘hypercyclic’ organization, the autocatalytic amplification inherent to such schemes can undeniably be observed in virtually every corner of the living world today. The *hypercycle* concept as such, therefore, appears well chosen, and the emergence of hypercyclic organic reaction systems has certainly played pivotal roles in the origins of life on Earth. Notably, the concept of “*catalytic closure*” in autocatalytic networks, as pioneered by Freeman Dyson [38] and Stuart Kauffman [39], is very much related to hypercyclic organization in general terms. Eigen and Schuster, however, concentrated their theoretical analyses on peculiar subclasses of hypercycle models, after having dismissed various more general alternatives—perhaps too lightly.

Most strikingly, the Eigen–Schuster concept is non-metabolic from the very outset. The *hypercyclic systems* considered therein primarily *connect self-replicative cycles* [37], consisting of tRNA-like *plus* and *minus* strands and primitive replicase enzymes encoded by the *plus* strands. The lasting coexistence of these subcycles is ensured by collective coupling in that each of these replicases assists one of the other replicators in a common hypercyclic loop. Everything else, however—even ribosome-like activities or any “*involvement of noninstructed, poorly adapted protein catalysts*”—is subsumed under auxiliaries “*in the form of ‘constant’ environmental factors*” [33]. The environment supporting such luxurious conditions was presumed from a rich *primordial soup* model, containing in free solution all the biochemical precursors required for RNA polymerization. It is these unrealistic assumptions that Jeffrey Wicken subjects to radical critique [36]. Other aspects of self-sufficient RNA predomination, too, have since been questioned as the “*RNA dreamtime*” scenario [40].

According to Wicken’s reasoning, the faithful replication of RNA alone has no selective value in any systems-related perspective. Moreover, replication systems in present cellular life bear no remnants of any hypercyclic organization of multiple and heterospecific replicases at all. Instead, such “*hypercycles are theoretical intermediaries invoked to bridge the gap between hypothetical replicators and biological organization. The merits of hypercycle theory must therefore be assessed in somewhat indirect fashion, by considering its assumptions and predictions in light of what we know about the likely chemical conditions of prebiotic evolution and the rationale of natural selection. ... A more realistic possibility is that life emerged through the co-evolution of nucleic acids and proteins within such proto-organizational structures as* [phase-separated and catalytic] *‘microspheres’. This approach is ‘organismic’, since molecules assume their relevance, and basis for selection, by virtue of their relationship to a ‘whole’ to whose stability or operation they are in some way relevant. ... Nucleic acids acquire genetic information within autocatalytic wholes to whose operation and proliferation that information proves relevant*” [36]. To concentrate on the heuristic value of hypercyclic organization at more general levels than looking at replication as an isolated phenomenon, it should be valuable to consider life as a tightly entangled system of *mixed metabolic-replicative hypercycles*.

As for quantifying the contents of total information accrued in biological organisms—or any other ‘*organization*’, for that matter—Wicken has pointed out some deeply embedded conceptual problems, which reach all the way back to life’s tentative emergence. “*The information of a paragraph is certainly not reducible to the Shannon complexity of letters on a page. The ‘succinct’ always delivers more information than the ‘rambling’. We can’t compute the information content of a treatise any more than we can compute the information content of an organism by a Shannon-complexity analysis of DNA sequences. Without inherited cytoplasmic controls DNA would have no way of expressing itself. Without the special environmental factors that have coevolved with living systems, those sequences wouldn’t even exist. Information content belongs irreducibly to systems—from ecosystems to works of literature*” [41]. 

To support this holistic approach to tentative origins of life from phase-separated organic conglomerates, Wicken refers to Sidney Fox’s experiments on “*proteinoid microspheres*” [42], which self-organize spontaneously when dry thermic condensates of pure amino acids are suspended in water. Quite influential several decades ago, though, Fox’s concepts have fallen into disregard upon heated controversies in the 1980s with the “*Nucleic Acid Monopoly*”, as Fox mockingly called his primary opponents [43]. Thereafter, the virtually uncontested primacy of nucleic acids over proteins has prevailed throughout origins-of-life research at large. Those personal controversies notwithstanding, the gist of Fox’s concepts deserves to be reincorporated into common scientific memory. 

The most interesting properties of Fox’s proteinoid microspheres may well be independent of the particular thermal anhydrous conditions he used to polymerize uncoded protein-like condensates from free amino acids at a large scale. Worth mentioning are (i) a partly biased (“self-coding”) incorporation of amino acids, (ii) versatile—still rudimentary—catalytic properties, (iii) phase-separated self-coherence due to hydrophobic interactions, and (iv) spontaneous assembly of membrane-like surrounding sheaths. As tentative model systems to conceive of efficient protein synthesis in watery media under prebiotic conditions become more sophisticated, and arguably more realistic [44,45,46,47], the general relevance of Fox’s observations on proteinoid microspheres is reemerging from a new perspective. And so are Wicken’s prescient considerations on the primordial coevolution of quasi-stochastic (uncoded) peptides and oligonucleotides into coded proteins and replicable nucleic acids, for that matter. In fact, in one of his later programmatic papers, Wicken tenuously suggests a plausible means to facilitate the watery emergence of prebiotic peptides—and catalytic microspheres, for that matter: “*One route for photoreceptors to discharge their energy in the prebiotic soup would have been by simple thermal dissipation. A more interesting route would have involved coupling to the phosphorylation of amino acids, ... which loses energy by polymerization to polypeptide ... In consequence, entropy ... is produced*” [6].

To conclude this central and important part, Jeffrey Wicken should have the last words in summarizing his critique of primordially hypercyclic replicators by himself. “[i] *Living systems are not hypercyclically organized, nor do they show any clues of hypercyclic ancestry. Nucleic acid replications are carried out by enzymes generally constructed for that purpose, rather than by populations of replicases each with their different strand specificities. ...* [except for viral co-option of a host replicative machinery] *we do not see* [the hypercyclic] *mode of operation in autonomous organizations. Hypercycles must therefore be justified as vehicles for moving from the prebiotic milieu to biotic organization*. ... [ii, Concerning adaptive commitment] *Can an RNA base sequence selected strictly according to phenotypic properties in replication be reasonably expected to somehow begin to act genetically—i.e, to serve as a template for protein synthesis? This notion does violence to conventional wisdom that adaptive commitments made under certain selective regimes constrain future adaptive possibility*. ... [iii, Concerning the chemistry of self-organization] *A first principle of molecular darwinism is that RNA replicators were the original, exclusive targets of prebiotic selection. If one adheres rigidly to this supposition, certain rules are set for the subsequent steps in life’s emergence.* [These rules impose hypercycles, but] *deny the theory much of the chemical resources and dynamics that were very likely available to prebiotic nature*” [36].

Further on, after discussing the alternative model of *phase-separated and catalytic microspheres*, based on Fox’s views, Wicken adds another reference to his support. “*Matsuno* [48] *advocates a ‘protohypercycle’ with mutual autocatalysis of proteinoid and nucleic acid and initial informing of the latter by the former. ... The protohypercycle might ... be a natural vehicle of autocatalytic evolution before the translation machinery had evolved sufficiently to reverse information flow to the present darwinian mode, and would be succeeded in evolutionary time by packaged hypercycles.* This *catalytic package does not rely on multiple replicases*” [36]. In a review quite favorable of Wicken’s critical considerations, Iris Fry recognized two opposing views among theorists on how to conceive of primordial biogenesis—the “*continuity thesis*” defended by a “*natural-law camp*” *versus* the “*happy-accident hypothesis*” implicitly assumed by the “*almost-miracle camp*” [49] (Table 1). Wicken’s views thus score high points on the ‘*continuist’* scale, well complemented by his holistic views on parallel continuities in present life of functionally coherent catalytic structure and informational backup from replicable genetic material. The integrative virtues of this holistic approach are further assessed in Section 5 and Section 6. To be more realistic, therefore, a comprehensive hypercycle theory of organismal coherence and autonomy should assume molecular complexes with general replicase activity in line with other molecular machines engaged in protein synthesis and/or RNA processing of various kinds, as well as metabolically and structurally important activities. Eventually none of this relates back to any single—ancestrally predominant—quasispecies of replicator sequences, as posited by the Eigen school [33,35], rather than to communal pools of quasi-stochastic sequences. It is from such randomized assemblies of prebiotic polymers, supposedly, that successive channelizing into structurally and functionally interacting clusters led to progressive coevolution of nucleic acids with proteins of gradually improving activities and specificities. More on these aspects is taken up below in Section 4. 

### 3.2. Wicken’s Mark on a Unified Theory of Evolution

The watershed divide between biological autonomy in a modern Darwinian sense and its ultimate connections to the physical world via its geochemical conditioning for life is iconically represented by the Last Universal Cellular (or Coenocytic) Ancestral State (LUCAS) [50,51], on which all the extant lineages of organismal life converge by retrograde extrapolation. To be sure, major families of proteins and their corresponding genes converge back on ancestral forms that preceded the LUCAS stage to variable extent. Thus, a lengthy formative era of proto-organismal relationships must have gone before the first successful bifurcation into recordable organismal lineages. While Jeffrey Wicken is remembered mainly for his thermodynamic approach to life’s beginnings, he also had firm views on how to connect biological evolution at large to physical conditions on thermodynamic terms. 

**Table 1:** Opposing views on life’s nature and beginnings.

	Two major conceptual frameworks	
	Continuity thesis	Happy-Accident hypothesis
Contestants	‘Natural-Law camp’	‘Almost-Miracle camp’
Signpost terminology	Life’s *emergence*, conceived as a continuous incremental process	A singular *Origin of life*, granted as a decisive improbable event
Temporal ordering of evolutionary agents	Metabolism before genetics; Stochastic peptides useful early on	Replication before metabolism;Stochastic peptides insignificant
Environmental foundations	Autotrophic organic syntheses around locally energized hotspots	Heterotrophic feeding on a rich primordial soup
Conceptual frameworks	Holistic duality of parallel continuity in coherent catalyst systems and digital genetic memory depositories	Genetic primacy over metabolic tools and mechanisms
Token synthesis	Rudimentary catalysts before digitally encoded, replicable templates	

In the 1980s, a group of dedicated scientists embarked on a movement to comprehend the regularities of Darwinian evolution on the basis of energy flows and thermodynamic forces or constraints [52], as generally discussed in [53,54]. These attempts were dubbed *‘New Synthesis’*, and Wicken took active part in that engagement, although with certain caveats [6,55]. His purist views on how to properly apply *entropy* and *information* in thermodynamic contexts—as opposed to the incongruous tradition of information theory—led him into rather divisive arguments with some of his colleagues, such as Daniel Brooks and Edward O. Wiley [1,7,56,57]. Wiley and Brooks [58,59] proposed that evolving biological species themselves are typical of systems that operate far from thermodynamic equilibrium and that evolution as such is an entropic phenomenon, where the so-called Shannon (informational) entropy steps in for energetic entropy of classical thermodynamics—as further discussed by David Depew [53]. These verbal disagreements about advanced evolution are only mentioned here in passing, since they are not of major concern to life’s primordial emergence. 

More substantially, Wicken emphasizes the continuity of major operational principles, both in the emergence of life on Earth and in its subsequent persistence. “*Ever since Alfred Lotka* [18] *began writing about energy flows as the basis of natural selection, there has been a thermodynamic paradigm in evolutionary theory that has coexisted with what we now loosely call neo-Darwinism. Lotka observed that selection will favor those organisms that, in pulling resources into their own services, also increase the energy throughputs of their ecosystems. Cooperation is built into the thermodynamic picture. ... Scientists operating in the neo-Darwinian tradition typically talk about the ‘origin’ of life, not its ‘emergence’. Life ‘happened’ somehow; then variation and natural selection did the rest. This isn’t enough. We need to define life so that its emergence can be connected with its operation*” [6]. 

The neo-Darwinian synthesis has brought impressive progress, but Wicken also saw possible pitfalls. “*August Weismann founded neo-Darwinism by demonstrating the continuity of the germ plasm and the derivative nature of the somatoplasm. This enormous accomplishment led, however, to an artificial division of organisms into ‘genotypes’ and ‘phenotypes’, and to the idea that genotypes were both logically and ontologically prior to phenotypes*” [6]. By taking these pitfalls to extremes, Richard Dawkins managed to divide the general public and scientific community alike [60,61]. As Wicken put it (extending on neo-Darwinism), “*its root metaphor—the idea that genes are ontologically prior to organisms—is increasingly troublesome. It has disposed thinking about organisms, and their connection with the rest of nature, in some very unprofitable directions—particularly the treatment of organisms as mediaries between genes and environments. From this, we get the ‘selfish gene’ motif* [60] *that interprets what organisms do as effectively caused by the survival payoffs to strands of nucleic acid*” [6]. For all the attention it has attracted, the ‘selfish gene’ metaphor itself is more of a provocative assertion than a scientifically valid generalization.

Biological organization is deeply entangled with its functionality [25,62]. “*All organizations have informed structural relationships which deliver certain functional ends*” [63]. This functionality relates to both organismal and ecological levels. Instead of considering “*organisms as ‘expressions’ of genetic programs*”, or “*mediators between genes and environments*”, the organism should have priority in this relationship—regarding “*genes as contributors to the system of internal relations by which the organism develops and maintains itself. ... Taking seriously the ecological condition of the genesis and maintenance of these relationships is no less important. Only through a ‘relational’ view of organism as a genetically-informed autocatalytic system sustained through thermodynamic flows ... can the perspectives of both ecologists and developmental biologists be accommodated*” [21].

These thoughts are well taken indeed and deserve to be further developed. Actually, the notion of “*organisms as mediators between genes and environments*” is not altogether wrong, but it cannot stand alone, of course. Genes, too, can be considered mediators—supplemental to the preceding remark on organisms. By providing a memory of previous successes on the metabolic front, genes are effective mediators between something that happened to have ‘worked’ in the past and upcoming repetitions of similar effect in the future. It is the recurrent stability of natural conditions in general that grants future success to any evolutionary strategy that is smart enough to take an average over past experience and to store it as a collective memory, thereby ‘anticipating’ that environmental conditions turn out about the same again on a regular basis. 

As for the tentative primacy of genes as selfish—or even autonomous—“*replicators*”, Dawkins’ vexing impact on “*the way ‘we’ think*” [61] (some of us, at any rate), is deceptively linked to an illogical contraption, which confounds the operational role of reagent (the gene to be replicated) with that of the active agents facilitating the mechanistic replication process. Amongst the myriads of organisms living today, there is not a single example or slightest hint that self-replicative genes do actually exist, or have ever existed in the first place. To be sure, modern genes are uniquely made up indeed in their *capacity of being replicated*, but all of these intrinsically depend on preexisting organized dynamic structures to accomplish the formidable jobs of genic replication on the one hand and cellular and/or organismic reproduction on the other. 

What Wicken wanted to achieve in his analysis of evolutionary theories was to formulate a direct link between natural selection—as a historically contingent process—and physically definable parameters of thermodynamic models. To continue the legacy of Jeffrey Wicken that “*life as a manifestation of the second law of thermodynamics*”, not the least in its subjection to Darwinian evolution, Eric Schneider and James Kay conclude that “*evolution, like ecosystems seems to select species and ecosystems that increase the global dissipation rate*” [64]. They base this claim on formal analyses focusing on steady-state phenomena, which stabilize autonomously far from thermodynamic equilibrium—provided there are utilizable gradients of energy, which last much longer than the dissipating phenomena under study. This is extending significantly beyond the “*dissipative structures*” considered by Ilya Prigogine before [65,66], which tended to return to equilibrium relatively fast. Schneider and Kay deliberately avoid the divisive issue of life’s purported relationship to *entropy* in maintaining their thorough eco-evolutionary approach. “*As ecosystems develop or mature they should increase their total dissipation, and should develop more complex structures with greater diversity and more hierarchical levels to assist in energy degradation ... Successful species are those that funnel energy into their own production and reproduction and contribute to autocatalytic processes thereby increasing the total dissipation of the ecosystem*” [67]. 

On the other hand, discussion of entropic concepts is not altogether obsolete in ecological perspectives, as shown in recent reviews on the *principle of maximum entropy production* [68,69,70], which applies to physics, chemistry and biology alike under far-from-equilibrium conditions [71,72]. Karo Michaelian, for example, has likened biological influence on the terrestrial hydrological cycle with “*life’s thermodynamic function*” [73] and conceptually linked the origin of life to increasing entropy production [74]. The controversial issue of relational order or intrinsic primacy between proteins vs. nucleic acids, however, which meant so much to Wicken’s views, is hardly addressed at all in these more general considerations.

To conclude these memorial sections, a note is in place about functional purpose in biology, which Wicken addresses in several articles. Purposive—“*teleological*”—concepts peculiar to living organisms have been discussed since ancient times, already by pre-socratic Greek philosophers [75]. In modern biology such concepts have been split into two components by Ernst Mayr, as summarized by Albert Eschenmoser: “*The general term ‘teleological’ (‘end-directed’ or ‘goal-directed’)* [was] *specified by Ernst Mayr* [76]. *The term ‘teleomatic’ is to be used when referring to a process which reaches an end state purely as the result of the operation of natural laws (e.g. gravitation or the second law of thermodynamics); alternatively, the term ‘teleonomic’* [77] *is to be used when referring to a process or a behavior which owes goal-directedness to the operation of a program (e.g., a genetic program)*” [78].

How then can ‘teleonomic’ means emerge from ‘teleomatic’ roots? In Wicken’s words, “*To be capable of propagation in the biosphere, certain reaction routes not only must be kinetically facilitated through appropriate enzyme catalysis; the specific catalytic activities involved also must be generated as integral parts of these thermodynamic flow patterns. Reaction routes capable of propagation therefore must be natural purposes themselves, that is, their own causes and effects. ... But the issue here is not the probability of particular scenarios having actually contributed to the emergence of life but rather the sense in which the emergence of natural purposes with genotype-phenotype organizations was as a general phenomenon promoted by the necessity of entropy production in stable patterns. Stable, propagating reaction pathways promote the cosmic end of entropy production and occur therefore as mechanisms in the biosphere’s overall thermodynamic evolution. Given the physical and chemical properties of abiotic proteins and nucleic acids, especially their mutually stabilizing interactions and the template-catalytic effects of nucleic acids on amino acid polymerization, the potencies of these molecule types certainly included the generation of a genotype-phenotype replicative mechanism, provided that a thermodynamic, natural-selective mandate for such a mechanism existed. And it did. Those reaction pathways that tended to propagate in the biosphere and to acquire their own autonomous stabilities were those that best exploited these chemical dispositions of proteins and nucleic acids to become synergistically involved in each other’s synthesis*” [75]. “*The general thesis is that all evolutionary and development processes can be understood as proceeding within certain ‘economies’, established by prevailing energy gradients and kinetic mechanisms for their utilization*” [63].

More recently, purposive, teleonomic concepts upon life’s emergence have been discussed again extensively by Addy Pross [79,80]. In earlier papers [3,81], he even cited Wicken’s work, but mainly for his “*two-tier approach to causation*”, and not for the major conclusions drawn therefrom. In fact, in most of his work Pross preferred to adhere to replicators-first presumptions. This commitment of his has only of late been softened, in that “*both template-directed autocatalysis and network formation may well have been critical elements in the emergence of life, most likely closely synchronized. That being the case, we would argue that the ‘replication first’—‘metabolism first’ debate, as a fundamental issue in the Origin of Life debate, may no longer be of real relevance, and should be replaced with a bridging ‘replication and metabolism together’ scenario*” [82]. This is no longer very much removed from Wicken’s views.

## 4. Integrative Perspectives

In this section I go into considerable mechanistic detail to point out incremental routes of connecting modern molecular complexity of life to likely stochastical beginnings. Impatient readers who might find such technicalities rather boring can advantageously proceed to the Concluding Remarks (Section 5) right away. On the other hand, any claims to the continuous emergence of life’s intricate complexity should be substantiated by reasonably plausible intermediary stages on its way from marginally self-sustainable growth to fully individualizable reproduction. Some of the particular technicalities suggested below are actually not part of mainstream thinking yet, but worth considering as tentative alternatives for further discussion. 

### 4.1. Circumstantials to Primordial Coemergence of Proteins and Nucleic Acids

How may stochastic peptides and oligonocleotides have started to cooperate at the very beginning?—Salient cues about the likely nature of this early coupling can be recognized as basic features preserved in nonribosomal peptide bond formation and/or ribosomal protein synthesis. There are good reasons to assume that the former mechanism is more ancient than the latter [83,84,85], paving the way for carboxyl activation of amino acids from thioester bonds to phosphoesters, and finally to ester linkage at one of the vicinal hydroxyl groups of ribose. In ribosomal protein synthesis this activating ribose moiety invariably belongs to the terminal adenosine at the canonical 3’-CCA end of tRNA. Ribose derivatives, therefore, are distinguished by their dual role—not only serving as versatile building blocks in nucleic acid polymerization, but also as convenient activating agents for peptide bond formation in aqueous medium. Phosphoryl amino acids, in particular, have also been proposed as a common link to primordial protein and nucleic-acid synthesis [86,87].

Since life on Earth strictly depends on water as its ambient solvent and reactive medium, efficient peptide synthesis under prebiotic conditions required some means of chemical amino acid activation to outweigh water’s hydrolytic tendency [44,45,46]. The energetic coupling can have relied on sunlight and/or geothermal gradients, and various models of primordial biogenesis have preferred one or the other of these potent energy sources over time [88]. For a considerable period, that is, some deep-sea hydrothermal vent scenarios prevailed in mainstream publications on this issue [89,90,91,92]. Of late, however, models of primordial photosynthesis assisted by colloidal photo-active minerals have become more realistic and attractive [93,94]—even to the abandonment of marine settings altogether, in favor of terrestrial hydrothermal fields, where phosphorus- and potassium-rich brines appear better suited to jump-start biogenic systems [95]. Notably, cytosolic P and K^+^ concentrations differ profoundly from those of seawater and may—in this regard—have faithfully conserved paleochemical conditions at life’s emergence from terrestrial (non-marine) environments [96,97]. 

If indeed sunlight provided the primal energy to drive the emergence of terrestrial life, the thermodynamic mechanism to facilitate this cause–effect relationship has likely worked as follows. During daylight hours the solid surface of the spinning Earth heats up most, because more radial energy is being absorbed and converted into heat than can be dissipated into space immediately; air and water bodies are heated as well, but convective currents redistribute much of the extra heat effectively. At night this cycle is reversed and solid surfaces cool down fastest. In the presence of photosynthetic life, however, not all the incoming light is converted into heat; a sizable fraction is deposited in organic carbon bonds instead so that surface heating is reduced considerably. Only part of this chemically stored energy is released by breakdown at night. The surplus contributes to biomass accumulation or is buried in sediments. In conclusion, therefore, Earth is a cooler place with autotrophic plant life present at its surface than it would be otherwise [2]. Also, such diversion of surplus energy into chemical storage—albeit inefficiently in the beginning—has likely driven primordial proto-life into existence. It took a lot of coevolutionary optimizing, though, to push its organizational infrastructure up to the level of individual cells that were able to reproduce as such (Section 4.2).

A surface-near, sunlight-exposed setting can have resulted in photochemical selection of base-paired nucleic acids, due to their remarkable overall resistance to the ultra-violet (UV) component of sunlight irradiation [98,99,100], together with hydrogen-bonded peptide systems for similar reasons [101,102]. The chemical selection for photo-stabile polymeric products must have started from relatively short sequences that happened to be stabilized as hydrogen bonded complexes, such as stem–loop or stem–elbow–stem motifs of RNA, α-helixes or β-hairpins of peptides, and mixed ribonucleoprotein (RNP) complexes with RNA-binding peptides. Although short peptides in general rarely form regular structures in aqueous solution, their potential to do so increases considerably by hydrophobic interactions between multiple nonpolar amino acid side chains. For that reason, simple aliphatic or amphiphilic peptides aggregate in membrane-like assemblies [103,104], or can form micellar nucleation centers for further interactions [105]. When mainly polar amino acids were subsequently added at the water-exposed interface around these hydrophobic cores, highly diversified surface patterns emerged, some of which happened to have cooperative or incidental system-wide effects—not unlike to what Sidney Fox had reported about his thermally produced proteinoid microspheres [42]. Others, too, have entertained similar concepts under different names, such as Alexander Oparin’s “*coacervates*” [106], Clair Edwin Folsome’s “*organic microstructures*” [107], or Krishna Bahadur’s “*Jeewanu*” [108], which are rarely considered in current mainstream theorizing about likely origins of life—still dominated by the intellectually appealing, yet operationally unrealistic postulate of lonesome genetic replicators’ primacy.

Following Jeffrey Wicken’s usage, it appears appropriate to apply *microspheres* as a general term for organic, phase-separated and catalytically active associations of prebiotic peptides, together with additional ligands, such as metal ions, phosphorylated metabolites, cofactors or various RNA molecules. In fact, rather simple amino acid sequences act as anion binding sites at so-called *nests*, particularly binding to phosphate groups and iron-sulfur centers [109,110,111], and simple cation binding sites occur about as frequently [111,112,113]. As such, these *phase-separated catalytic microspheres* were central to a progressively self-organizing peptide–cofactor world [114,115], from which genetic replicators of transiently double-stranded RNA eventually emerged, primarily as protein-enhanced and protein-reinforcing back-up devices providing processive protein-generating facilities as well as robust memory storage for that capacity. To add a contemporary example of experimental research on *phase-separated microspheres*, Mikhail Kritsky’s group has used polymolecular systems consisting of flavoproteinoid pigment molecules from thermally rearranged amino acids, aggregated in aqueous medium to model the abiogenic photo-activated synthesis of ATP [116].

For all his rational critique of the neo-Darwinian tenet “*that RNA replication was the only, or the most powerful, competitor for nucleotide and energy resources in the prebiosphere*” [36], Wicken did not contribute any particular theory as to how a reliable replication mechanism might have emerged in the first place. Yet under any scenario of primal biogenesis, the mechanistic details of how genetic replication could have come about are quite important issues. As viewed from an *RNA World* perspective, various proposals have been made regarding the piecemeal assembly of RNA by processive triplet-transferring ribozyme replicases [117,118,119,120], from which peptide-transferring proto-ribosomes may have developed later on. Under Wicken’s coevolutionary view, however, it appears more likely that such ratcheting RNA replicases, if they ever existed, developed in parallel with proto-ribosomes—and even spliceosomes, conceivably. These composite RNP machines presumably emerged from the same communal pool of quasi-stochastic uncoded peptides and oligonucleotides that resided in the *phase-separated catalytic microspheres* of the prebiosphere.

This coevolutionary primordial scenario comprises a multi-component and multi-cycle adaptive enterprise, where overall efficiency is gradually optimized. The diametrically opposed principles, which cannot both be maximized simultaneously, are dissipation of energy gradients on the one hand and accumulation of macromolecular organic matter on the other. As for the (almost) universal code for mRNA-directed ribosomal protein synthesis, it has been empirically assessed that this system is highly optimized indeed [121,122,123]. Furthermore, to reach such a level of optimality effectively from self-organizing competition, the winning system must have cooperated on a large communal basis [124], as had been conjectured before by Carl Woese for the organizational nature of “*The Communal Ancestor*” [125]. By the same token, presumably, the optimization of early replication—first of RNA, and then for DNA as well—has sprung from such a large communal pool of many small cooperative components. The composite cellular RNA polymerases—of eukaryotes in particular—and the complex “*replication factories*” for genomic DNA in all three domains of organismal life bear witness to such a legacy. In contrast, small efficient template-specific replicases of viral RNAs appear highly streamlined by reductive evolution, adapted to the minimalistic and hijacking life style of their parasitic vectors [126]. As viewed from this perspective, the phage-specific Qβ RNA replicase, which has been instrumental in Eigen’s and Schuster’s hypercyclic modeling of hetero-specific replicators [31], cannot reasonably be considered representative of early evolution in large communal systems—originating from innumerable quasi-stochastic sequences.

### 4.2. Biogenic Compartmentalization and Random-Event Evolution

In challenging the Eigen–Schuster model of replicative hypercycles in prebiotic evolution, Wicken’s critical commentary [36] was actually preceded by another critique of Carsten Bresch and coworkers from a slightly different angle [127,128]. Both these approaches overlap on various aspects of prebiotic compartmentalization, and the differences appear significant. While Eigen’s group responded promptly to Bresch’s concerns [34], Wicken’s critique seems to have gone unnoticed. Both being physicists by training, Eigen and Bresch would dispute on a ‘similar wave length’, seeing no reason to draw into question the general *primacy of genetic replicators*. Yet Wicken’s holistic—‘*metabolist*’—approach was alien to both of them alike. 

In short, Bresch, Niesert & Harnasch argue in two directions. (i) They seriously question the evolutionary stability of the “*triple functionality*” of all the RNA members engaging in the hypercyclic relationship proposed in the model. If this relationship cannot be upheld against mutational degradation, it should not have emerged in the first place. (ii) As an alternative model, they suggest that some means of “*packaging*” were necessary early on to get self-organizing principles under way. In such packages—however loosely these were tied together—only one type of replicase would be necessary for the replication of all strands of RNA available in a package [127]. Wicken supported the latter argument as well, but his main opposition ran deeper still.

As mentioned before, in the abstracted Eigen–Schuster model there was nothing tangible between the beautifully symmetrical reciprocity of primal replicators, tRNA-like adaptors and templated replicases in their functionally interconnected relationship and a rich ‘*prebiotic soup*’ all around this idyllic scenario. Even ribosome-like entities to fabricate the perfectly mutualistic replicases were considered constant ingredients of a benevolent yet relatively stagnant environment. In Wicken’s words to summarize the “*realistic hypercycle*” of [33] by quoting from [37], “*Initially, compartmentation was undesirable:* ‘Organization into cells was surely postponed as long as possible. Anything that interposed spatial limits in a homogenous system would have introduced difficult problems for prebiotic chemistry. Constructing boundaries, transporting things across them, and modifying them when necessary are tasks accomplished today by the most refined cellular processes. Achieving analogous results in a prebiotic soup must have required fundamental innovations’ [37]. *But these are precisely the emergent properties of the catalytic microsphere, which require no appeal whatever to selective payoff* ” [36]. As further discussed below, the phrasing of this quote might well have been a little more precise to be consistent. Eigen’s pivotal point is that cell-like outer *boundaries* should be undesirable too early, and other means of compartmentalization are not necessarily included in his statement. Wicken, too, has a valid point in his objection, but only inasmuch as the *boundary-independent* packaging effects in microspheres are concerned.

It is only fair to note, though, that Eigen and Schuster cautiously kept open some back door for thoughtful awareness. “*In our discussion so far we have done perhaps some injustice* [*sic*!] *to experiments simulating primordial, template-free protein synthesis, which were carried out by S.W. Fox and others* [129]. *It was the goal of our studies to understand the early forms of organization that allowed self-reproduction, selection, and evolutionary adaptation of the biosynthetic machinery, such as we encounter today in living cells. Proteins do not inherit the basic physical prerequisites for such an adaptive self-organization, at least not in any obvious manner as nucleic acids do. On the other hand, they do inherit a tremendous functional capacity, in which they are by far superior to the nucleic acids. Since proteins can form much more easily under primordial conditions, the presence of a large amount of various catalytic materials must have been an essential environmental quality. Research in this field has clearly demonstrated that quite efficient protein catalysis can be present under primordial conditions. Interfaces deserve special recognition in this respect. If covered with catalytically active material they may have served as the most favorable sites of primordial synthesis. The restriction of molecular motion to the dimensions of a plane increases enormously the efficiency of encounters, especially if sequences of high-order reactions are involved*” [33]. 

Evidently Wicken and Eigen set out to understand the same set of problems—“*the early forms of organization that allowed self-reproduction, selection, and evolutionary adaptation of the biosynthetic machinery*”—but their respective means of comprehension turned out to be quite different. Both of them were prominent proponents at either side of the breach between metabolist and geneticist views on what came first [3,81], a coherent and energized protometabolism or genetic replicators in their own right. Whilst Eigen’s view remained fixated on replication and tried to grasp its roots in isolation, denying the surrounding ‘soup’ any infrastructural significance, Wicken was convinced that this approach was bound to fail. The main argument for this denial has been as follows. Replication as such has no system-promotive value in itself. It would merely deplete the “*rich prebiotic soup*”—had that ever existed—for energy-rich precursors, thus driving the soon-to-be-overadapted replicators to abrupt extinction [36]. Arguing from a holistic stance, instead, Wicken insisted on a system-promotive cause from the very beginning. His supposition that life’s emergence hinged upon some energetic charging of the prebiosphere did not aim at any prebiotic soup in free solution, rather than at structured foci of nucleation for the accretion of progressively more organic macromolecules. Even Manfred Eigen made it a point in the beginning that “*Evolution must start from random events*” [30], alas he all too soon allowed this knowledge to drop out of sight—in favor of the fictive notion that life stood no chance before it could faithfully replicate some basic information. 

For Wicken the crucial question was not primarily what makes life propagate by self-similar replication, rather than what makes it tick more basically. Whatever it is that does make life tick—could this capacity manifest itself in a rudimentary way on the basis of randomly assembled organic polymers? Wicken was convinced it could, and Fox’s microspheres—as model systems—were key to this conviction, especially if similar kinds of phase-separated catalytic aggregations of prebiotic peptides and associated ligands could be photo-energized to harvest sunlight for *in situ* synthesis of ever more organic building blocks, high-energy bonds and further oligomeric products. These kinds of aggregates fulfilled some of the criteria for prebiotic *packages*, *sensu* Bresch *et al*. [127,128], even though they were not yet comparable to bona fide cells in any way. 

First of all, these packages need not have had any distinct boundary, since their coherence was primarily based on covalent bonding within individual polymers and mutual affinity binding by mostly agglutinative hydrophobic interactions, rather than on passive containment by closed vesicles of any kind. In topological terms, hydrophobic connectivity comes about at three different levels, as characterized by fractional values of spatial dimensionality around zero (point-like), one (quasi-linear) or two (quasi-planar)—for, respectively, {0} hydrophobic cores of globular enzymes; {1} extended scaffolds, such as the fibrous material of a ‘*nuclear matrix*’ in eukaryotic nuclei, to which the chromosomes appear to be anchored at multiple sites; and {2} spherically closed envelopes of membrane-like consistency with hydrophobic aggregation along the central plane and polar end groups all along the outer faces. Fully compact structures {3} can occur as well as oily droplets or solid precipitates, but have little relevance as such for biogenic organization in general. Only the closed vesicular configuration of fully evolved bi-layer lipids qualifies as a hermetic outer boundary, and Eigen was probably right in assuming that such an obstacle should not come in too early in prebiotic evolution. It is a pity that Wicken in his objection to this point did not fully realize the aggregational potential of focal cores and fibrous scaffolds, which are not liable to undesirable boundary effects early on. As pointed out in Table 2, only peptides and proteins are capable of suiting such aggregational needs at all three structural levels, comprising the most versatile category in this regard. Knotty single-stranded ribozymes are of a limited import, and double-helical DNA fibers serve no structural purpose in functional infrastructure. This ambiguity among various packaging aspects is but one example for being misled by the popular impression that a bacterial cell resembles the most primitive compartment conceivable at the dawn of life. *Cell* and *compartment* are by no means synonymous terms. Accordingly, the phase-separated microspheres, *sensu* Wicken, should have retained a high tendency to confluence, exchanging macromolecules to some extent and thereby satisfying the communal optimizing potential posited by the Woese school [124,125]. Not inconceivably, many or most of them were spread out in two-dimensional patches at various mineral surfaces [51], in the uppermost pore space of grainy sediments. Short distance in the third dimension and little boundary effect of such arrangement would allow small molecules easy access to and from the stream of percolating fluid.

Strangely enough, there is a curious air of unreality about the geneticists’ early simulations of how to generate life-like systems from a virtual singularity of informational entities. In the Eigen–Schuster models it is but a single ‘*quasispecies*’ in nucleic acid *‘sequence space*’ [35] that is supposed to underlie the common root of all later genes and chromosomes. In the package model of Bresch and Niesert [127,130] the founding source is not much more diversified, since more than three different genes per package would destabilize the system under the general assumptions made in their model. In other words, all those assumptions cannot approximate reality in any reasonable way. The clonally constrained propagation of replicable genetic entities cannot as such be considered an *evolutionarily stable strategy* (ESS) at the earliest stages of prebiotic evolution. Only by changing those assumptions could Vetsigian, Woese & Goldenfeld [124] arrive at more realistic computer simulations, allowing for remarkable optimization of multiple components and parameters in a large communal system with many collective interactions.

To paraphrase a passage quoted from Simon Black [11] above (Section 2.2), the prebiotic ecosystem of catalytic microspheres could run virtually all the basic reactions necessary for life’s physical persistence—*only at rates that were billion-fold slower*. Most of the ingenious innovations collected in cellular life later on were primarily selected and optimized for their capacity to increase overall metabolic reaction rates. Although the mainstream—*geneticist*—faction among evolutionary thinkers may find it awkward even to consider, this metabolic imperative for phenotypic selectability should also hold up for the emergence and perfection of accurate template replication. From the *metabolist*’s vantage point of energetic and material flow rates, considering prebiotic microspheres as interactive networks, their low initial overall efficiency could only be improved by gradual changes at many interaction nodes simultaneously. This is a tantalizing nonlinear optimization problem with many variables. 

**Table 2:** Dimensional modalities of biogenic hydrophobic structuring.

Dimension	~0	~1	~2
Morphology	micellar, point-like	linear, fibrous	flat, vesicular
Peptides/proteins	++	++	++
RNA/DNA	+	(++)	–
Lipids	(+)	–	+++

### 4.3. The Coding Problem—a Matter of Entangled Hyper-Coupling

If, indeed, prebiotic peptides stood for much of the initial, however low, catalytic activity of microspheres, the low rate of unaided peptide bond formation could be improved by interaction with ribotides in several stages, (i) at the level of amino acid activation, (ii) making stochastic peptide bond formation a processive process for several amino acids in a row, (iii) discriminating between non-polar and polar amino acids to facilitate the formation of hydrophobic cores, and (iv) discriminating between more and more individual amino acids to complete the full canonical table of the genetic code.

Chemical activation by phosphorylated carbohydrates was a convenient option. If, as mentioned above, partly base-paired oligoribotides were physically selected as particularly photo-stabile compounds in surface-near, sunlight-exposed settings [97,98,99,100], prebiotic oligonucleotides and peptides could functionally meet at the pivotal node of chemical amino acid activation. *Nota bene*, these UV-resistant oligoribotides were not yet selected for accurate replicability in any way, nor for any particular base pair sequence. Random addition of terminal residues would perfectly suffice initially, and the folding back of short single strands could subsequently utilize base pair complementarity in forming a stem–loop hairpin [131,132]. Somewhat more complex stem–elbow–stem structures and the tRNA cloverleaf motif, as well as related configurations, could likewise arise by such quasi-stochastic mechanisms. 

Several key mediators of RNA-assisted protein synthesis can conceptionally be related back to structural configurations of this kind, i.e. to potentially quasi-stochastic roots. This is most obvious for the tRNA cloverleaf, which itself is assumed to have arisen from single-stem hairpins. Next in line is the heart of the larger ribosomal subunit, comprising the peptidyl transferase center (PTC), which supposedly has evolved from a dimerized pair of stem–elbow–stem motifs [133,134]. This funnel-shaped dimer presumably had (and still has) the important role to hold both termini of the charged and interacting tRNAs in position, thus shielding the high-energy bonds between tRNA and amino acids from the hydrolytic potential of surrounding water, whereas the transfer reaction itself is solely catalyzed by the ribose moieties carrying the energized bonds [5,135,136]. Around this protective funnel then, a ratcheting device has assembled, which still forms the most ancient heart of the smaller subunit [137]. The two subunits have since coevolved together as an RNP machine consisting of numerous ancient RNA motifs and many interspersed peptides, cooperating as a dynamic scaffold with processive action. Consensus has it that for quite some time the evolving protoribosome was still engaged in the synthesis of more or less stochastic peptides, with little preferential sequence information. Nevertheless, the processivity achieved by this innovation must have meant a lot for speeding up the rate of making that kind of prebiotic peptides. 

For as long as the charging of hairpin stems in tRNA-like adapters was unspecific regarding which amino acid to attach to a particular RNA sequence, of course, the resulting peptide sequence had to remain stochastic. At that stage already it was possible that additional non-structured RNAs—perhaps of repetitive sequence—were employed to keep the loops of charged tRNAs in place during the reaction. From such a stability-enhancing brace, mRNAs could evolve later on [138], together with specific charging for diversifying tRNAs. Along this path the first bias toward rudimentary coding rules was likely introduced as distinguishing criteria between non-polar and polar amino acids [139,140]. Such measures could generate the hydrophobic cores and scaffolds of growing microspheres preferentially, *i.e.*, at higher frequencies than expected by chance from stochastic incorporation of polar and non-polar residues alike. The full table of the canonical—and virtually universal—genetic code would then be gradually established in a multi-component optimizing process resulting in a chain of similarity-based codon reassignments [124,141,142,143,144].

These possibilities for rate improvement must have meant a lot for prebiotic evolution, but production rates of initially uncoded peptides comprised only one side of the coin. As recognized and pointed out by Christian de Duve, selection by differential molecular survival was perhaps yet more important early on, when unaided peptide formation was just beginning and still rather ineffective [145]. His model assumed that various multimeric compounds expressed a wide repertoire of rudimentary catalytic activities and that some lytic system degraded these multimers again, unless those compounds were differentially protected. Such exemption could result from protective assembly into tightly packed molecular configurations, including ligand binding during catalyzed reactions. The hydrophobic interactions in the cores and scaffolds of prebiotic microspheres, of course—as assumed by Wicken and others—would likewise grant such protective shielding from hydrolytic breakdown. 

In this multi-objective optimization framework, functional RNA replicases were but one among many enzyme activities potentially useful for system maintenance and growth at large. The faithful replication of long-chains RNAs from one end to the other is not a trivial affair, and it is doubtful whether such activities in the modern sense—involving the alternate synthesis of full-length *plus* and *minus* strands—existed at all during much of prebiotic evolution. Instead, effective synthesis of prebiotic RNA could initially have been confined to functionally useful *plus* strands exclusively. Two different regimens to accomplish just this have so far been discussed in the literature: Stuart Kauffman’s closed networks of *collectively autocatalytic sets* [39,146]—as adapted to RNA-rearranging ribozymes or RNP complexes—and Harry Noller’s *duplicator-mediated RNA duplicase* [120].

There is, in fact, some enigmatic triplet pattern pervading various RNAs of ancient evolutionary origin: RNY > RNR > YNY > YNR [147,148]. More often than not, this pattern has been discussed in the context of a translatable triplet code at the beginning of ribosomal protein synthesis—even to include the implication that ribosomal and other non-translated RNAs showing this pattern were originally translated as well. The alternative hypothesis, however, could be more generally relevant that essentially all prebiotic RNAs at some stage were assembled from a “*foodstuff*” pool of *trinucleotides*, which in turn were being synthesized according to that biased pattern by a special subset of a Kauffmanian *autocatalytic network*. In such a scenario, therefore, the appearance of a full set of *minus* strands—as intermediates for *bona fide* replication—had to await the perfection of the genetic code, when coded proteins could step in as faithful replicases, directional helicases and other accessory factors. Notably, the universal CCA trinucleotide carrying the activated amino acids is still added secondarily to residual tRNA sequences [149].

Of all the intricate relationships in biological organization, the emergence of coded protein synthesis is often said to represent the most enigmatic hindrance to comprehending life’s emergence in terms of physical principles, which is exceedingly difficult to overcome. David Abel has even gone so far as to seriously question the feasibility of such a quest on ontological grounds [150,151]. In an overbearing tone he denounces rational attempts to understand part of the problem by refining physicodynamic theories as ‘wishful thinking’ for so long as no counter-example can be produced of “*non trivial, unaided spontaneous optimization of formal function by truly natural process*” to falsify his self-proclaimed “*null hypothesis*” that “*physicodynamics alone cannot organize itself into formally functional systems requiring algorithmic optimization*” [150]. I do not share this resigning attitude toward scientific efforts to narrow down conceptional gaps by piecemeal approximation from within.

The simple fact that we do not yet fully comprehend all of life’s secrets is no reason to believe that we cannot come closer to this fictive goal in the future. That the ultimate goal may, in principle, remain fictive forever does not disqualify the scientific value of the knowledge and comprehension to be gained in narrowing the gap. Erwin Schrödinger, whose quest for life’s peculiar orderliness stood for the opening passages of this essay, is most renowned for his highly successful wave functions. Presumably this is the way for further pursuit. Many aspects of life relate to oscillations or resonance phenomena. Even purely physical wave systems, such as so-called rogue, freak or killer waves on the open ocean, where all the interacting molecules are small and uniform—are not yet fully understood in all their details, but progress continues to be made [152,153]. Similar, although far more complex than simulating ocean waves, progress can also be made in understanding life-like self-organization, where numerous longer and more versatile molecules react on one another.

Upon reading Abel’s self-assertive creed “*Life manifests innumerable formalisms that cannot be generated or explained by physicodynamics alone*” [150], I am tempted to insert a more moderate “*cannot* yet (!)”. As the example of freak waves was supposed to indicate, we are far from having comprehended the full potential of *physicodynamics* yet. Abel’s insistive opinion that the complexity of life is completely out of reach for physicodynamic comprehension is not really compatible with scientific modesty. He is perfectly right, of course, that all the dissipative structures hitherto analyzed in non-equilibrium physicodynamics are still a far cry off from truly life-like self-organization. Significantly though, none of the more tractable fluid-dynamic model systems involves organic macromolecules of any kind. 

In other words, a more thorough understanding of the physicodynamic potential of biogenic or biomimetic macromolecules—originating from stochastic roots—is needed badly. The Eigen–Schuster replicative hypercycle model was an ambitious attempt in that direction. The major shortcoming was, however, that this model had no direct bearing on the emergence of coded protein synthesis, which actually should be considered the very heart of coevolutionary coupling between proteins and nucleic acids. Ribosomes, tRNAs and charging enzymes are at least as important to life’s persistence as are template-dependent replicases.

Any self-consistent future paradigm should be founded on the concept that—in an environment composed and energized appropriately—both peptides and nucleic acids could arise spontaneously in short and random sequences, albeit at rather low rates initially. Their possible short-length *sequence spaces* were essentially filled up at the beginning. Both these polymeric chain molecules have different yet periodic backbone structure, bearing aperiodic side chain residues. Variation in their side residues, therefore, comprises their respective sequence space. The dynamics of further evolution depended on a probabilistic matrix of correlation coefficients, describing the slightly biased influence of preexisting sequences on the net generation of new molecules, as adjusted for interrelated rates of sequence- and context-dependent degradation. In the course of subsequent evolution, sequences of individual molecules became longer, but the corresponding inflational sequence spaces were no longer fully occupied. Selection in sequence space must have acted according to certain *pruning rules*, the stringency of which increased with time. Can we expect to understand these rules from any channeling constraints?

Owing to the different properties of peptides and nucleic acids in aqueous surroundings, the overall matrix of dynamic correlation coefficients bears certain asymmetric features from the beginning. Whilst peptides and protein bring in their intrinsic potential for hydrophobic packaging, together with the stabilization of various catalytic pockets at the surface of the hydrophobic cores, RNA contributes its remarkable base-pairing complementarity. Together they are much stronger—more durable and versatile—than is either kind alone. Those sequences that bind together better than others resist lytic breakdown and/or washout into the surrounding sink of open waters, whereas single, unstructured molecules are being decimated. In accordance with de Duve’s suggestion [145], this is the most reasonable initial pruning rule to start the first population of RNP-dominated microspheres on a stochastic basis. The more this primordial population grows, the higher becomes the potential for stochastic fluctuations in both protein and nucleic acid sequence space, which in turn are the raw material for resonance enforcement of covariationally better utilization of the energizing gradient that drives the organic system in the first place. Multiple cycles of such reenforcement comprise the basis for coevolutionary success. Vetsigian, Woese & Goldenfeld [124] have pioneered the productive simulation of such optimizing cycles. 

In mathematical phrasing, the end result of the canonical genetic code represents a unidirectional mapping function from one sequence space into another. At face value this deterministic mapping is merely a formal framework, which—to be brought into effect—needs a physical mechanism of many functionally structured and cooperative components. As Vetsigian &c put it, “*The genetic code is a probabilistic map ... between codons and amino acids. The map is probabilistic because the charging of tRNAs with particular amino acids and the decoding of codons through competition of tRNAs are probabilistic molecular events*” [124]. In this language, evolution of the now fairly stringent map from feeble beginnings amounts to narrowing down the probability distribution of codons from being nearly uniform in the initial stochastic fluctuations toward the rigid pattern we can see today.

The many functional and interactive components—one or more multi-purpose replicases included—are perpetually being replenished in an enormous collectively autocatalytic network, composed of multiple subcycles. In such general terms, therefore, the organization of living matter is based on integrated hypercycles. Pivotal to its evolution has been the formal alignment of two structurally independent sequence spaces by resonance interference between certain stochastic fluctuations in either space. The functional output of this resonance has been assessed in terms of growing efficiency of beam splitting for the energizing gradient, diverting more energy into overall self-similar organic synthesis and less energy into dissipation as ambient heat. This functional coupling between interactive subsets, drawn from two disparate macromolecular sequence spaces, is genuinely unique to life. In due respect for Eigen’s ingenuity, I am tempted to denote this uniquely complex kind of functional entanglement as *hyper-coupling*. 

## 5. Concluding Remarks

Jeffrey Wicken was a deeply rational and independent thinker, who was not shy of challenging some of the brightest minds of his time on what he considered an ingeniously conceived hypothesis that happened to be incongruous with fundamental principles of nature. Wicken saw his mission in connecting the exquisitely complex relationships in the living world with more readily comprehensible principles pervading the physical world from long before the onset of life on Earth. He fully realized that such a conceptual connection had to be founded on thermodynamic and kinetic considerations, calling for a better understanding of statistical end energetic principles as applied to biogenic macromolecules. Moreover, he strongly argued in favor of the holistic principle that some structural assemblies of catalytically active organic molecules had to start growing coherently, before rudimentary genic replicators could gain any selective advantage in reinforcing more effective growth of such organic assemblies. To repeat some key remarks already included in a longer quote above (Section 3.1.), “*Scientists operating in the neo-Darwinian tradition typically talk about the ‘origin’ of life, not its ‘emergence’. Life ‘happened’ somehow; then variation and natural selection did the rest. This isn’t enough. We need to define life so that its emergence can be connected with its operation*” [6]. Yet more pointedly, the essence of this passage culminates in similar considerations by Iris Fry, who claims “*that the ‘almost miracle’ view* [that life’s origin happened by some unique low-probability transition] *implies in fact, a creationist position*” [49].

From such premises of the “*continuity thesis*” (*sensu* Fry, Table 1), Wicken argued that the replicational hypercycle concept, as specifically devised by Eigen and Schuster, was untenable as an explanation for the emergence of life. Its basic tenet that genetic replication has to manifest itself before most other metabolic functions lacks selective value under primordial conditions and, thereby, remains conceptually unconnected to a preexisting physical world. In the present essay, I have described the background for this profound criticism as a prototypical example for a deeper lying dualism between *genetics-first* and *metabolism-first* positions on the *origins-of-life* agenda. Whether replicators or metabolism emerged first is actually one of the oldest and most divisive controversies about tentative origins of life [81,154,155,156,157], and the conceptual primacy of replicators over metabolic tools is still considered mainstream by many investigators. Has Wicken thus argued in vain? It rather seems his critical paper [36] was simply overlooked. Only 11 citations can currently be retrieved by Google Scholar, and none of these is from any dedicated proponent of a genetics-first scenario. 

This archetypical controversy of dualistic concepts reverberates in the bio-philosophical discourse under various guises, from ‘mind over matter’ and cell nuclei *vs*. proto- or cytoplasm to genotypes over phenotypes. It is tempting to merge Wicken’s insights and Woesean wisdom about these matters as follows. The energetically charged prebiosphere had to consolidate a collectively functioning phenotype, before any individualistic genotypes could stand a reasonable chance of escaping, so as to make a living on their own. From there on, these individual organisms had to engage in relentless competition (as well as in symbiotically collaborative ecological relationships) with ever more diversifying other genotypes.

As even Addy Pross now recognizes the merits of a “*replication-and-metabolism-together scenario*” [82], the following framework appears to be more realistic. The origins of life are founded on three major roots, in this order of temporal, functional and logical priorities: a lasting energetic gradient on the pristine Earth between the radiating solar source and the sink of outer space; self-accreting networks of prebiotic macromolecules that happened to work together slowly; and an emerging archive to let the consolidating network remember how it actually had worked in the preceding period. To begin with, both the consolidating catalytic network and the nascent working memory were rather ‘fuzzy’ systems. By resonating and falling into sync with one another they became subject to coevolutionary optimization. In recognizing the gist of these relationships, Jeffrey Wicken was clearly ahead of mainstream thinkers at his time. Two other—now little remembered—‘*giants he stood on the shoulders of*’ were Sidney Fox and Simon Black; Sidney for his insights into the catalytic potential of large collections of uncoded protein-like aggregates, Simon for his concepts of hydrophobic stickiness among amphiphilic organic macromolecules as the subtle and dynamic core of biogenic self-organization. Their common, complementary legacies are worth remembering when the history of science is recorded. As for Jeffrey himself, he added a strong, holistic emphasis of structural coherence and persistence, together with functional utility in living matter, to this conceptual triad.

## 6. Epilogue—Added in Proof

### 6.1. From “Thermodynamic Controls and Stability” to “Kinetically Controlled Stability” [3,79,80,81,82]—or “Lost in Translation” [158]?

Appreciating the constructive input of two expert referees, I have focused the manuscript on several issues, but the following suggestion could not be readily accommodated within the original framework. That insightful comment indeed raises a number of important points, which happen to touch on both conceptual and communicational issues. I shall, in turn, discuss and motivate several aspects of dissenting opinion, as seen from the perspectives presented in the current paper.

**Anonymous**: “*The manuscript would benefit from a tighter connection to contemporary thinking. Wicken, while realizing that factors other than thermodynamics had a role to play in explaining biological organization, was struggling to put his finger on how those other factors could be integrated into a comprehensive theory. Wicken stressed thermodynamics to be the driving force for emergence and evolution but failed to convince the wider community. Clearly something more was needed. In fact it is that failing that led Wicken’s ideas to slip from the minds of contemporary workers. But in recent years, an alternative view, that kinetics plays an inordinate role in the emergence and establishment of biological complexity, and has to be integrated into the thermodynamic view, has begun to make inroads into this long-standing problem. See, for example: Eschenmoser* [159]*, Pross* [82] *and Pascal* [160]*. The central issue—how animate emerged from inanimate—is not simply encapsulated in the ‘replication-first/metabolism-first’ debate, as implied in the manuscript. It goes a lot deeper. Some recognition of those newer ideas that discuss kinetic imperatives (‘kinetic stability’ as opposed to ‘thermodynamic stability’) would add further weight to* [this] *essay.*”

### 6.2. What Is It That Actually “Led Wicken’s Ideas to Slip From the Minds of Contemporary Workers”?

As is often the case, ‘it takes two to tango’, and either partner can be liable for a failure to fall into sync. Other prescient thinkers have suffered a similar fate before. When Gregor Mendel disclosed his now famous hybridization results on garden peas, he met his contemporary audience of classical botanists ‘off beat’ entirely, whilst zoologists could not see much relevance to their subject, and biologists in an overarching sense did hardly exist. I also think that Wicken’s contemporary audience at large was not fully prepared to listen in on his objectives. Moreover, Wicken happened to publish most of his theoretical considerations in the conjectural form of ‘*Research proposals*’—hopefully to be substantiated in the future by further refinement—rather than as comprehensive ‘*Research results*’. More often than not may other researchers, of individualistic mind, show reluctance in adopting an unfamiliar project that is not of their own making. To be more specific, Wicken’s conception of thermodynamics comprised a science of stochastic fluctuations among large numbers of interacting particles together with energy fluxes, to the extent that the characteristics of these interactions could be formalized by mathematical equations—quite independent of whether these equations happened to be solvable or not. This far-reaching concept cannot easily be substantiated by experimental chemistry under artificially constrained conditions.

### 6.3. Can Wicken’s Stressing of Thermodynamic Imperatives be Rightfully Equated with Pross’ and Others’ Concept of “Thermodynamic Stability”—as Opposed to “Kinetic Stability”?

I now describe why the answer to this rhetorical question ought to be “Not at all.”—When Wicken embarked on his quest to chart direct connections between living organisms and the physical world, he resorted to *thermodynamics* as a quite general part of *statistical physics*. To be sure, certain principles derived from this branch of physics have been successfully applied to chemical reactions as well, for which a more limited terminology has developed to suit the most pressing and specific needs of chemistry. The well-established technical terms of *thermodynamic control* and *thermodynamic stability* for chemical reactions bear witness of this limited tradition in their being coupled to equilibrium conditions exclusively, as opposed to *kinetic control* and *kinetic stability*. The overarching *thermodynamics* branch of physics, on the other hand, deals with statistical regularities and relationships at all levels of particle–energy interactions, including dynamic flows of energy and matter—irrespective of whether equilibrium has been realized, or is being approached for that matter. The booming literature on *non-equilibrium thermodynamics* fully attests to this statement. Albert Eschenmoser stringently defines these technical terms of *statistical chemistry* for us in his comments already cited above [159], as applying to the assessment of product concentration under pair-wise competitive constraints. More colloquially speaking, the decisive difference can also be characterized as “*reactions under kinetic control (the fastest reaction wins)* [*vs*.] *thermodynamic control (the most stable product wins)*” [161]. Eschenmoser then makes it bluntly clear that mere shifting of terminology as such does not solve the enigma of life’s emergence.

“*While it has become a truism that life requires an overall chemical environment that is far from equilibrium, kinetic control of life processes is a requirement for the overall process, but parts of it can proceed under (partial) thermodynamic control. What has been paramount to the origin of life with respect to the dichotomy of thermodynamic versus kinetic control is the central role of catalysis in imposing kinetic control on structural changes of a chemical environment held far from equilibrium by kinetic barriers*” [159].

Jeffrey Wicken was perfectly aware, of course, that life in general is characterized by its vital dependence on lasting energized gradients to grant life’s maintenance and persistence quite far from thermodynamic equilibrium. He was equally aware of the crucial role of catalysis in life’s emergence. That is exactly why he saw so much instructive value in the work of Sidney Fox on *catalytic proteinoid microspheres*—taking this experimentally tractable example for a suitable case to model the eventual transition from rudimentary catalysis by quasi-statistically varied structure to progressively higher effectiveness, as mediated by informationally instructed/encoded biocatalysts. 

As for semantics, Wicken used ‘*dynamic*’ and ‘*kinetic*’ in numerous relationships, but more in a physical sense than the chemistry-specific usage of these attributives—in particular: “*Kinetic mechanisms are means for processing energy, operating at the microscopic level of the causal hierarchy in service of the second law*” [63]. This is not quite the same as the wording of Pross, where both these related qualifiers can even become entangled as ‘*dynamic kinetic stability*’ [3,79,80,81,82] or similar composite expressions. As viewed from Wicken’s overarching perspective, not particularly obliged to this decidedly chemical terminology, the two kinds of dynamic control or stability, as defined by the chemical terms here stated, are very real and important to keep in mind, but considering but one of those as being “more thermodynamic than the other” is somewhat absurd in the physical sense and certainly of little help in broader understanding across boundaries of language or culture between divergent fields of reference.

On the other hand, a substantially novel approach of experimental chemistry, which should become increasingly relevant to origins of life research in the future, has been introduced by Powner, Gerland & Sutherland [47]. They remarkably arrived at activated pyrimidine ribotides by a novel, unanticipated pathway. Phosphorylated forms of the naturally occurring RNA constituents cytidine and uridine accumulated as the *most stable products*, and did so from—by conventional standards of synthetic chemistry—rather ‘dirty’ experimental conditions. These researchers started from a mixture of four rather simple organic reagents, utilized phosphate for its multiple potentials, first as versatile catalyst and buffer system in several early reactions, and then as chemical reactant at a later step. Finally they used prolonged UV irradiation, both to convert part of the initially formed cytidine into uridine and to effectively quench numerous undesirable side reactions. Most surprisingly, the crucial conformation of ribose did not even occur as a free precursor, but only later inside a composite intermediate.

Taken at face value, this digressive foray into experimental work may appear to add just another piece of ‘boring detail’ to an all too complicated story, but it is brought here for a better reason. Those ground-breaking experiments demonstrated convincingly that mixed chemistry is feasible and relevant; they also showed that caring for detail does matter a lot. It should be interesting indeed to include amino acids as primary reactants in similar ‘single-pot’ reaction schemes, together with the purging effect of prolonged UV irradiation. The potential range of even the narrowly defined *thermodynamic control* (the most stable product wins) has by far not been exhausted in currently tractable theory or practical experiments, and serious studies outside the still prevailing ‘RNA World’ umbrella have barely yet begun. The purging UV effect, in particular, could have imparted ‘self-cleaning’ properties to localized ‘geochemical reactors’, which not only channeled the synthesis of organic metabolites toward the *most stable intermediary products*, but also led further toward the *most stable polymers*. Whilst selection for the most UV-resistant metabolites, under phosphate-catalyzable reaction conditions, would substantially reduce the sheer multitude of biochemically possible reactions [154], the most UV-stable polymer associations could contribute to de Duve’s selection model [145].

### 6.4. How Deeply Does the Notorious Replication-First/Metabolism-First Debate Touch on the Central Issue?

Were it only for the academic question whether one or the other intrinsic property of modern life had any temporal advantage at early-Earth conditions, the public audience at large might not care less. As this debate lingers on, however, it should be stressed that the opposing catchphrase identifiers reside over more deeply reaching differences (Table 1). Traditional *replication-first* proponents expressed little concerns about the mechanistic origins of replication, other than presuming that activated genetic precursors were freely available in the ‘*primordial soup*’ environment. That ‘soup’ was considered devoid of catalytic activity to start with, and all the metabolic catalysts emerging later on had to be ‘invented’ *de novo* by the emerging genetic system. Their opponents from the *metabolism-first* school, however, are seriously in doubt whether such a rich-soup scenario has ever existed. Instead they assume that essentially all the organic compounds necessary and sufficient to make organic macromolecules at any scale were synthesized *in situ* at energized hotspots in a suitably heterogeneous mineral-dominated environment. The first generations of macromolecules were subsequently formed around these hotspots, and only those that physically stayed together in a kind of phase-separated film-like hydrogel emulsion could contribute to further prebiotic evolution, where optimization of selectable catalytic activities was the main evolutionary incentive. 

To start with, mere physical binding of potential substrates to potential catalysts could influence the rates of polymerization and/or decay significantly enough to influence their initial evolution. Most notably, in this scenario replication itself is considered but one of many catalytic activities emerging and being optimized progressively and simultaneously. The overall decision about which catalysts were to be favored over others was guided by a more and more self-constrained network of all the reactions that collectively contributed to a form of balanced growth in the envisaged molecular hydrogel community. This network of biogenically useful reactions is also known as protometabolism. Essential branches of this network must have been in place quite early on, however fuzzily they began to stand up against the background noise from stochastic fluctuations.

Initially, stochastic fuzziness prevailed at every level, from the type and number of metabolically useful biochemical reactions over the compositional sequence space of corresponding catalysts and the complementary sequence space of corresponding genes to the operational mapping function of the emergent code between these disparate sequence spaces and the replicases to replicate the genes. To optimize this hyper-network of coevolving reactions, catalysts, templates, coding rules and processive aids, no single component could reduce the stochastic fuzziness associated with its own formation and functional specificity significantly faster than could many other factors, the specific fuzziness of which had repercussions on the possible degree of constrainability—the evolutionary loss of ‘fuzziness’—for the first one. In the course of this coevolutionary optimization process, the rudimentary first-generation catalysts with rather ineffective but broad-spectered activities were gradually replaced by veritable enzymes, encoded more and more accurately, and specificities were narrowed down accordingly. Hence, many of these next-generation catalysts were not, in fact, ‘invented’ *de novo* by the emerging genes, but were steadily selected for being able to cooperate with a working system that was already in place. The gradual transition from *uncoded* to gene-*encoded* protein synthesis can fittingly be described as a ‘*genetic takeover*’. This term implies that contingently active prebiotic catalysts were replaced by genetic innovations, which led to more readily renewable and more efficient catalysts of corresponding metabolic function.

### 6.5. Can Anything Reach Deeper than to Initially Stochastic (Thermodynamic) Fluctuations?

Few, if any, scholars will now question that multi-component coevolutionary optimization of coded protein synthesis and corresponding nucleic acid replication has been close to the heart of emerging terrestrial life [124]. The continuing enigma lies in how this bimodal optimization problem has come into existence. Tracing back such coevolution of bimodal systems coupled in ‘lockstep’ usually reaches a certain bifurcation point at the transition from an indistinctively unitary preceding state. As the principles of *bifurcation theory* clearly are relevant for the emergence of biochemical complexity at later times [162], it should be rewarding indeed to apply similar analyses to the initial translation–replication problem. To the best of my knowledge, extensive efforts have not yet been made along these lines. Whilst the Wicken–Black approach to thermodynamically sound biogenesis quite naturally implies the existence of that pivotal bifurcation point, it is less clear how the Prossian takeover by “dynamic kinetic controls” should mechanistically relate to that critical transition. 

As for the physical nature of present life, the holistic duality of functional bodies and digital genetic memory depositories is of paramount significance and interest. The entire DNA of multiple human genomes—taken as purified molecules in isolation—is ‘nothing but’ dead organic chemistry, only being subject to relentless decay until it eventually vanishes in thermodynamic equilibrium. Also, deprived of functional processing systems for template-encoded gene expression and replication, as properly coupled to energized metabolism, all its highly praised contents of genetic information become worth nothing. Extending backward from all the presently living organisms to long before their last common ancestral life form, two unbroken lines of continuity have existed on parallel tracks for 3–4 billion years: the cytoplasmic track of functional machinery and the informational track of digital instructions encoded in the genome.

### 6.6. Resetting Focus on the Balance Sheet Reshuffled

A thoughtful, informative and highly recommendable book on the holistic nature of life has been ‘composed’ by Denis Noble [163]. Not least, his witty and sobering critique of Richard Dawkins’ ‘*tour d’esprit*’ on the selfishness of genes is worthy of note. Sending “The Gene” on a self-replicational ego trip alone is stripping all genes in general of their cooperative virtues. This leads to much distorted views of life’s essential nature and characteristics. Even viral genes do not simply replicate on their own, but must functionally cooperate with other genes of the same genome; and for accomplishment of their ‘selfish’ propagation, the entire virus particles depend on the functional catalytic infrastructure of live host cells. This reciprocal interdependence between catalysts and genes goes back unbroken to a stage of ‘fuzzy’ indeterminateness around the tentative primordial bifurcation point mentioned above. Before that stage, rudimentary catalysts amongst polymeric molecules of stochastic composition were the primary organic agents on the primordial scene. Some of these catalysts may well have consisted of single-stranded RNA, whilst others were prebiotic peptides. Thereafter, the first gene-like sequences availed themselves as a kind of ‘memo stickers’ to bias synthesis in favor of certain peptide catalysts over other molecules of less selective value. It must have taken quite a while to collect and coordinate sufficiently many memo pads to make for a comprehensive archive—containing and being able to maintain all the instructions necessary to generate entire individualizable cells anew. To emphasize the roles of active players at the initial transition, the so-called “*Metabolism before Genetics*” paradigm can be rephrased in complementary terms as “*Rudimentary catalysts before digitally encoded, replicable templates*” (Table 1, bottom).

Jeffrey Wicken was keenly aware of the double-track nature of living matter, and he did not hesitate to argue against leading proponents of the gene-centered paradigm at his time. Holistic reasoning of this kind is what attracted me most to Wicken’s writing. His insights are highly worth remembering.
